# Super-resolution microscopy reveals the number and distribution of topoisomerase IIα and CENH3 molecules within barley metaphase chromosomes

**DOI:** 10.1007/s00412-023-00785-8

**Published:** 2023-01-31

**Authors:** Ivona Kubalová, Klaus Weisshart, Andreas Houben, Veit Schubert

**Affiliations:** 1 Leibniz Institute of Plant Genetics and Crop Plant Research (IPK) Gatersleben, D-06466 Seeland, Germany; 2 Carl Zeiss Microscopy GmbH, D-07745 Jena, Germany

**Keywords:** CENH3, Centromere, Chromatin, *Hordeum vulgare*, Photoactivated localization microscopy, Structured illumination microscopy, Super-resolution, Topoisomerase IIα

## Abstract

Topoisomerase IIα (Topo IIα) and the centromere-specific histone H3 variant CENH3 are key proteins involved in chromatin condensation and centromere determination, respectively. Consequently, they are required for proper chromosome segregation during cell divisions. We combined two super-resolution techniques, structured illumination microscopy (SIM) to co-localize Topo IIα and CENH3, and photoactivated localization microscopy (PALM) to determine their molecule numbers in barley metaphase chromosomes. We detected a dispersed Topo IIα distribution along chromosome arms but an accumulation at centromeres, telomeres, and nucleolus-organizing regions. With a precision of 10-50 nm, we counted ~ 20,000-40,000 Topo IIα molecules per chromosome, 28% of them within the (peri)centromere. With similar precision, we identified ~13,500 CENH3 molecules per centromere where Topo IIα proteins and CENH3-containing chromatin intermingle. In short, we demonstrate PALM as a useful method to count and localize single molecules with high precision within chromosomes. The ultrastructural distribution and the detected amount of Topo IIα and CENH3 are instrumental for a better understanding of their functions during chromatin condensation and centromere determination.

**Supplementary Information:**

The online version contains supplementary material available at 10.1007/s00412-023-00785-8.

## Introduction

Determining the ultrastructures of cell nuclei and chromosomes and quantifying their molecular components are required to understand the dynamics of such basic biological processes as transcription, replication, and cell division. Often, fluorescence dyes are used to label the protein of interest. In contrast to classical fluorescence microscopy techniques, super-resolution microscopy such as structured illumination microscopy (SIM) and single-molecule localization microscopy (SMLM), including photoactivated localization microscopy (PALM) are well suited to achieve ultrastructural imaging by breaking the diffraction limit of light (Schermelleh et al. 2019; Khater et al. 2020). In PALM, fluorophores are excited in such a way that only one molecule of many within the diffraction-limited spot is in its “On” state and can reach precisions up to ~10-40 nm (Betzig et al. 2006).

Meanwhile, SIM, implemented in different microscopic platforms, has been widely used in cell biology (Heintzmann and Huser 2017). It was applied to scrutinize chromosome scaffold proteins (Poonperm et al. 2015) and unveiled the localization of the replication protein A on chromosome axes during meiotic recombination in mammals (Yoon et al. 2018). Moreover, SIM revealed that the mammalian genome, in interphase nuclei, is organized into functional chromatin domains of ~200-300 nm in diameter (Miron et al. 2020).

PALM uncovered dynamic clusters of cohesin and the insulator protein CTCF (Hansen et al. 2017) and contributed to elucidating the mammalian higher-order chromatin structure (Nozaki et al. 2017). PALM was also successfully applied to bacteria to monitor transcription (Stracy and Kapanidis 2017). The development of a three-dimensional (3D) assay for transposase-accessible chromatin-PALM enabled to study chromatin domains and genome topology changes in single cells (Xie et al., 2020).

In plants, SIM was used to visualize chromatin and associated proteins in interphase nuclei and condensed chromosomes (Schubert 2014, 2017; Nemečková et al. 2019; Shi et al., 2019; Zelkowski et al. 2019; Kubalová et al. 2020; Schubert et al. 2020; Municio et al. 2021). Besides, SIM is useful to investigate other cell structures (Schubert 2017) such as microtubules of *Arabidopsis* and *Medicago* (Komis et al. 2014, 2015a, b, 2017, 2018; Vavrdová et al. 2019; Tichá et al. 2020).

On the other hand, SMLM, like PALM studies, is still limited in plants. Schubert and Weisshart (2015) determined the number of RNA polymerase II molecules in differentiated *Arabidopsis* nuclei by PALM. Besides, PALM was used to analyze microtubules and microtubule-associated proteins in *Arabidopsis* root epidermal cells (Vavrdová et al. 2020).

Recently, we compared different super-resolution microscopy methods and proved their superiority over diffraction-limited fluorescence microscopy to analyze chromosomal chromatin. The achieved lateral SIM resolution of ~100 nm and PALM localization precision of up to ~10 nm demonstrated that the combination of both techniques provides a comprehensive overview of Topoisomerase IIα (Topo IIα) localization in barley metaphase chromosomes at the ultrastructural level (Kubalová et al. 2021b).

Topoisomerases are involved in transcription, DNA replication, and chromatin organization (Björkegren and Baranello 2018; Meijering et al. 2022; Pommier et al. 2022). Topo IIα is a dimeric enzyme (~175 kDa in human) owning catalytic and non-catalytic functions. The former depends on ATPase activity, whereas the latter relies solely on the C-terminal domain (CTD) (Fukui and Uchiyama 2007; Edgerton et al. 2016). The catalytic function ensures that supercoiled or catenated DNA becomes resolved via DNA strand passage. Topo IIα introduces double-strand breaks into dsDNA, thus allowing other DNA fibers to pass through. Afterward, the break becomes sealed without any loss of DNA information. This action is important for several biological processes such as DNA replication, transcription, chromosome condensation, and segregation (Nitiss 2009). Although CTD is dispensable for decatenation (Dickey and Osheroff 2005), it is essential for the targeting of Topo IIα in mitotic chromosomes (Lane et al. 2013). First, Topo IIα resolves inter-chromatid entanglements, then it generates intra-chromatid entanglements to promote thickening. Only the latter process requires the CTD (Shintomi and Hirano 2021). The majority of Topo IIα concentrates in the inner centromere and is associated with the control and activation of cell cycle checkpoints as demonstrated in human, mouse, and muntjac cells (Coelho et al. 2008; Lane et al. 2013; Gomez et al. 2013, 2014; Nielsen et al. 2020).

Besides, Topo IIα localizes in chromosome arms. Topo IIα was found as a component of the chicken and human mitotic chromosome scaffolds (Earnshaw and Heck 1985; Earnshaw et al. 1985; Samejima et al. 2012; Chu et al. 2020). The application of light and electron microscopy uncovered twisted double-stranded protein scaffolds in both human metaphase chromatids. These scaffolds are composed of alternating Topo IIα enzymes, condensins, and kinesin family member 4 (KIF4) proteins (Ono et al. 2004; Samejima et al. 2012; Poonperm et al. 2017; Chu et al. 2020). The importance of Topo IIα was demonstrated via its depletion, disrupting the scaffold structure (Poonperm et al. 2015).

Although most data originate from mammalian research, it was reported that plant Topo IIα acts in mitotic and meiotic recombination (Singh et al. 2004). In onion (Zabka et al. 2014) and tobacco (Singh et al. 2017), Topo IIα is involved in cell cycle regulation and removes meiotic bivalent interlocks in *Arabidopsis* (Martinez-Garcia et al. 2018). Thus, Topo IIα possesses several roles while residing on mitotic chromosomes, each requiring a precise location and number of available molecules.

Centromeres, occurring as distinct primary constrictions (monocentromeres) or distributed along chromosomes (holocentromeres) (Schubert et al. 2020), are fundamental for correct chromosome segregation during mitotic and meiotic cell divisions. Thus, they secure the proper distribution of genetic material into daughter cells. Tandemly repeating DNA sequences of different lengths among species are typical for these regions. In contrast to non-centromeric chromatin, most centromeres contain a specific histone H3 variant, termed CENH3 (or CENP-A) (Palmer et al. 1991; Talbert et al. 2002; Cleveland et al. 2003; Ali-Ahmad and Sekulić 2020). In most eukaryotes, CENH3 specifies the position of a proteinaceous complex, the kinetochore, where spindle fibers attach pulling the chromosomes towards both daughter cells (Musacchio and Desai 2017). Vast numbers of proteins residing at the centromere and in the kinetochore detect the fidelity of the spindle fiber attachment and eventually trigger cell cycle checkpoints (Cleveland et al. 2003; Hindriksen et al. 2017). Thus, the vital function of centromeres based on the presence of a certain CENH3 amount is required.

The monocentromeres of most plant species, like rye, barley, *Aegilops speltoides*, and *Cuscuta japonica* are determined by CENH3, and their ultrastructures were analyzed by SIM (Wanner et al. 2015; Schubert et al. 2016, 2020; Oliveira et al. 2020). SIM revealed that barley CENH3 is localized mainly in the interior, rather than at the surface of the monocentromeres. Only a low amount is present in the pericentromeres. Barley encodes two CENH3 variants, αCENH3 and βCENH3, interacting with a fraction of *Cerebra*, a centromeric retroelement (CR)-like repeat, and besides with a GC-rich centromeric satellite (Houben et al. 2007; Sanei et al. 2011; Schroeder-Reiter et al. 2012). α and βCENH3 colocalize and form together two distinct globular intermingling structures at the primary constriction of mitotic and meiotic metaphase chromosomes (Ishii et al. 2015; Wanner et al. 2015).

SIM has also been used to quantify the relative amount of immuno-labeled CENH3 during the mitotic and meiotic cell cycles of rye (Schubert et al. 2014). However, SIM investigations cannot determine the absolute number of molecules. The specific number and localization of proteins are required to understand their function in the chromatin organization of interphase nuclei and during cell divisions. Moreover, these data are necessary to improve polymer simulations (Câmara et al. 2021; Kubalová et al. 2021a) explaining chromatin condensation along chromosome arms and at centromeres.

In this work, we show that PALM/SMLM is a useful method to count and localize single molecules with high precision. We applied SIM and PALM to localize and quantify the number of Topo IIα and CENH3 molecules based on immuno-labeled somatic barley metaphase chromosomes. The observed accumulation of both proteins within centromeres shows their need to arrange plant centromeres. Furthermore, Topo IIα is present along chromosome arms probably necessary to condense chromatin.

## Results

### Topo IIα occurs dispersed at arms but accumulates at centromeres, telomeres, and NORs of barley metaphase chromosomes

**Fig. 1:**
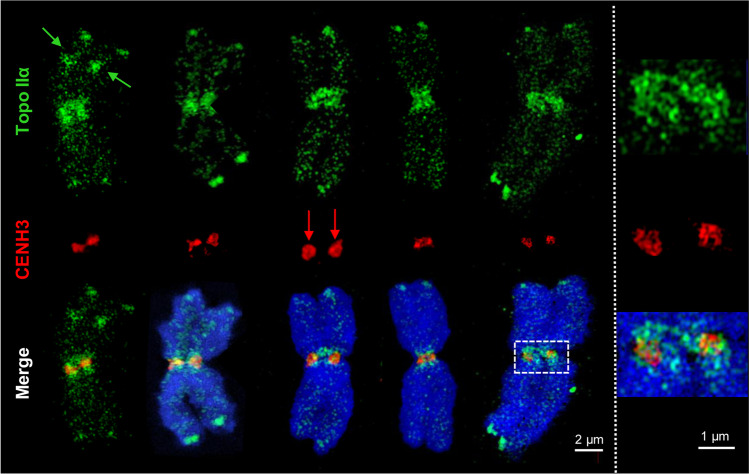
Colocalization of topoisomerase IIα (Topo IIα) and CENH3 detected by 3D-SIM. The Topo IIgp13 antibodies used in this experiment show a similar pattern as the Topo IIrb12 antibodies on barley metaphase chromosomes (see Fig. 2). CENH3, sometimes forming ring-like structures (red arrows; Schubert et al. 2016), is embedded within the Topo IIα labeled centromeric chromatin. The satellite chromosome 6H (left) shows Topo IIα labeling also at the NOR regions of both chromatids (green arrows). The telomeres of all chromosomes accumulate Topo IIα with varying intensities. The enlarged pericentromeric region of the right chromosome indicates the intermingling of Topo IIα and CENH3-labeled chromatin. The merge exhibits besides Topo IIα (green) and CENH3 (red) the whole chromosomes stained with DAPI (blue)

**Fig. 2:**
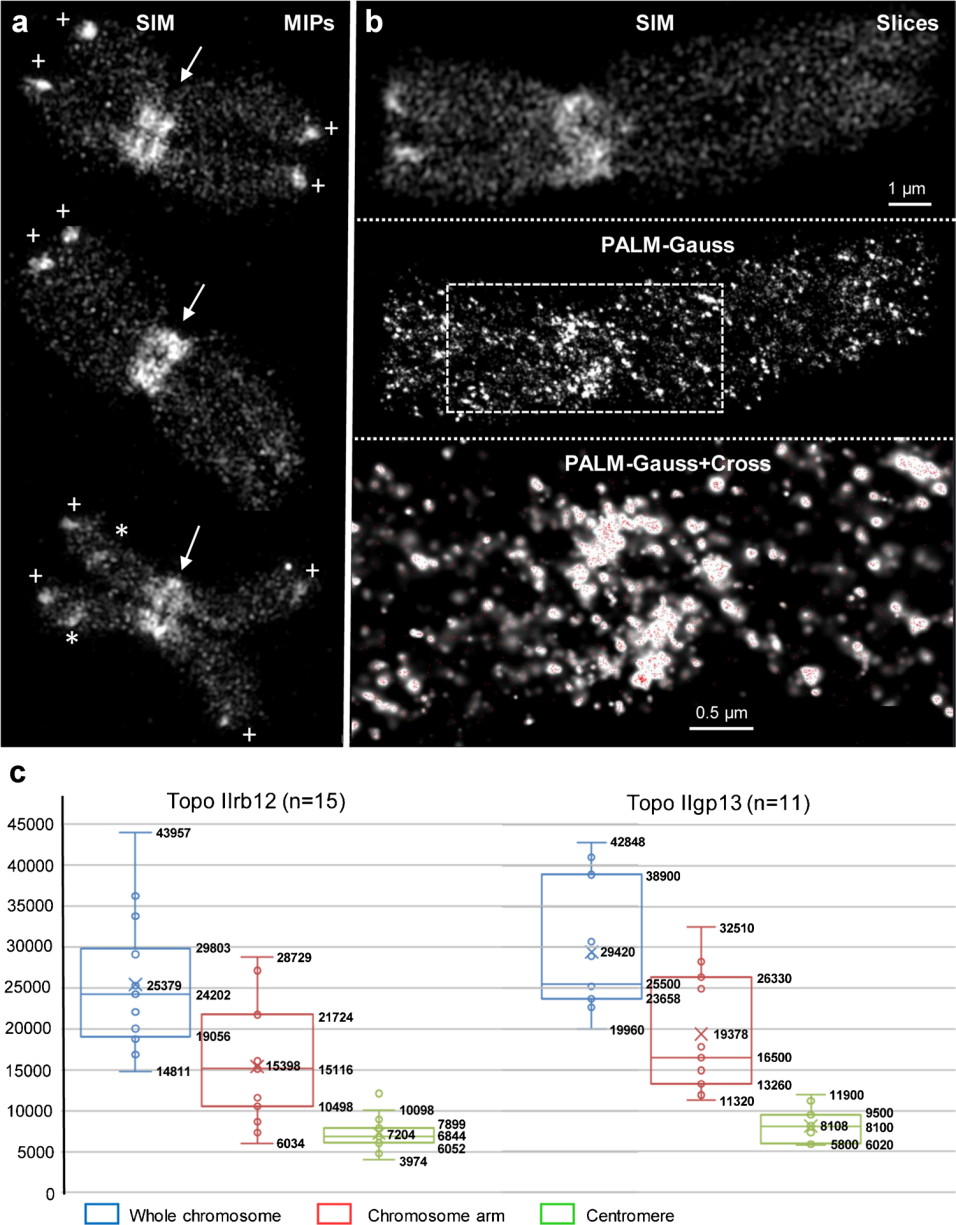
3D-SIM and 3D-PALM of using Topo IIα in different barley metaphase chromosomes visualized by using Topo IIrb12 Alexa488-labeled antibodies. **a** Maximum intensity projections (MIPs) of 3D-SIM image stacks show the accumulation of Topo IIα at all (peri)centromeres (arrows), at some subtelomeres (crosses), and NORs (asterisks) of satellite chromosomes. Topo IIα is homogeneously distributed along all chromosome arms. **b** Single slices of 3D-SIM (top) and 3D-PALM image stacks acquired consecutively from a chromosome showing the Topo IIα accumulation besides at the (peri)centromere only at the short arm telomere. The enlarged region (bottom) within the dashed rectangle of the chromosome visualized via PALM-Gauss (middle) shows additionally the localization of single Topo IIα molecules (red crosses). **c** Boxplots representing the Topo IIα molecule number variability in 15 and 11 chromosomes analyzed by 3D-PALM using Topo IIrb12 and Topo IIgp13 Alexa488-labeled antibodies, respectively. Numbers indicate lower whisker, 25% quantile, median, mean, 75% quantile and upper whisker

To analyze the distribution of Topo IIα at the ultrastructural level, we stained flow-sorted barley chromosomes with specific antibodies and applied 3D-SIM. Both Topo IIα peptide antibodies raised in rabbits and guinea pigs (Topo IIrb12 and Topo IIgp13, respectively) (Kubalová et al. 2021b) revealed similar enzyme distribution patterns on metaphase chromosomes (Figs. 1, 2; Movies 1, 2). Figure 1 shows the labeling pattern of five different chromosomes. In all of them, Topo IIα occurs in a network-like manner. Movie 1 demonstrates by running through a 3D-SIM image Z stack of the chromosome shown in Fig. 2b that Topo IIα is homogeneously distributed at the surface and within chromosome arms. This becomes also obvious by rotating the same image stack (Movie 2). The satellite chromosomes 5H and 6H (left chromosome in Fig. 1 and bottom chromosome in Fig. 2a) exhibit an accumulation of Topo IIα within their nucleolus organizing regions (NORs). Besides, Topo IIα is accumulated at some telomeres with varying intensity as demonstrated especially at the bottom arms of the second and fifth chromosomes. The enzymes concentrate within the pericentromeres as visible on all chromosomes shown in Figs. 1 and 2.

To figure out whether Topo IIα and CENH3-positive chromatin colocalize at centromeres, the chromosomes were labeled with CENH3-specific antibodies in addition (Fig. 1). Similar as shown previously in *Arabidopsis* and cereals (Schubert et al. 2016), CENH3 labels cluster- or ring-like ultrastructures as shown on the third chromosome of Fig. 1. Topo IIα and CENH3 detecting the inner centromere are differently positioned but intermingle among each other (enlarged region of the fifth chromosome in Fig. 1). Movie 3 visualizes the spatial Topo IIα and CENH3 distribution and colocalization in the same rotating 3D-SIM image Z stack.

### Twenty-eight percent of the ~20,000–40,000 Topo IIα molecules per chromosome localize within the pericentromere

To determine the absolute Topo IIα molecule numbers per chromosome, we performed 3D-PALM (Fig. 2b). The PALM imaging confirmed the molecule distribution patterns visualized via SIM. Both different Topo IIα antibodies delivered similar molecule numbers indicating the reliability of the antibodies and the 3D-PALM method.

The chromosome in Fig. 2b showed after 3D-PALM in the Gauss visualization mode a similar Topo IIα distribution as via 3D-SIM. The red crosses within the Gauss mode indicate the exact position of the localized and counted molecules within the enlarged centromeric region of a single PALM slice (Fig. 2 bottom). Running through the PALM-Gauss image Z stack exhibits the spatial distribution of Topo IIα (Movie 4).

The number of Topo IIα molecules (~20,000-40,000) varied highly between the 15 and 11 chromosomes analyzed by Topo IIrb12 and Topo IIgp13 Alexa488-labeled antibodies, respectively (Fig. 2c). On average, ~27,400 Topo IIα molecules are present within whole chromosomes, ~17,400 along arms, and ~7700 around centromeres. That is, ~28% of molecules are accumulated in the (peri)centromeric region (Fig. 2c).

### Topo IIα enzymes surround ~13,500 CENH3-containing nucleosomes at centromeres

**Fig. 3:**
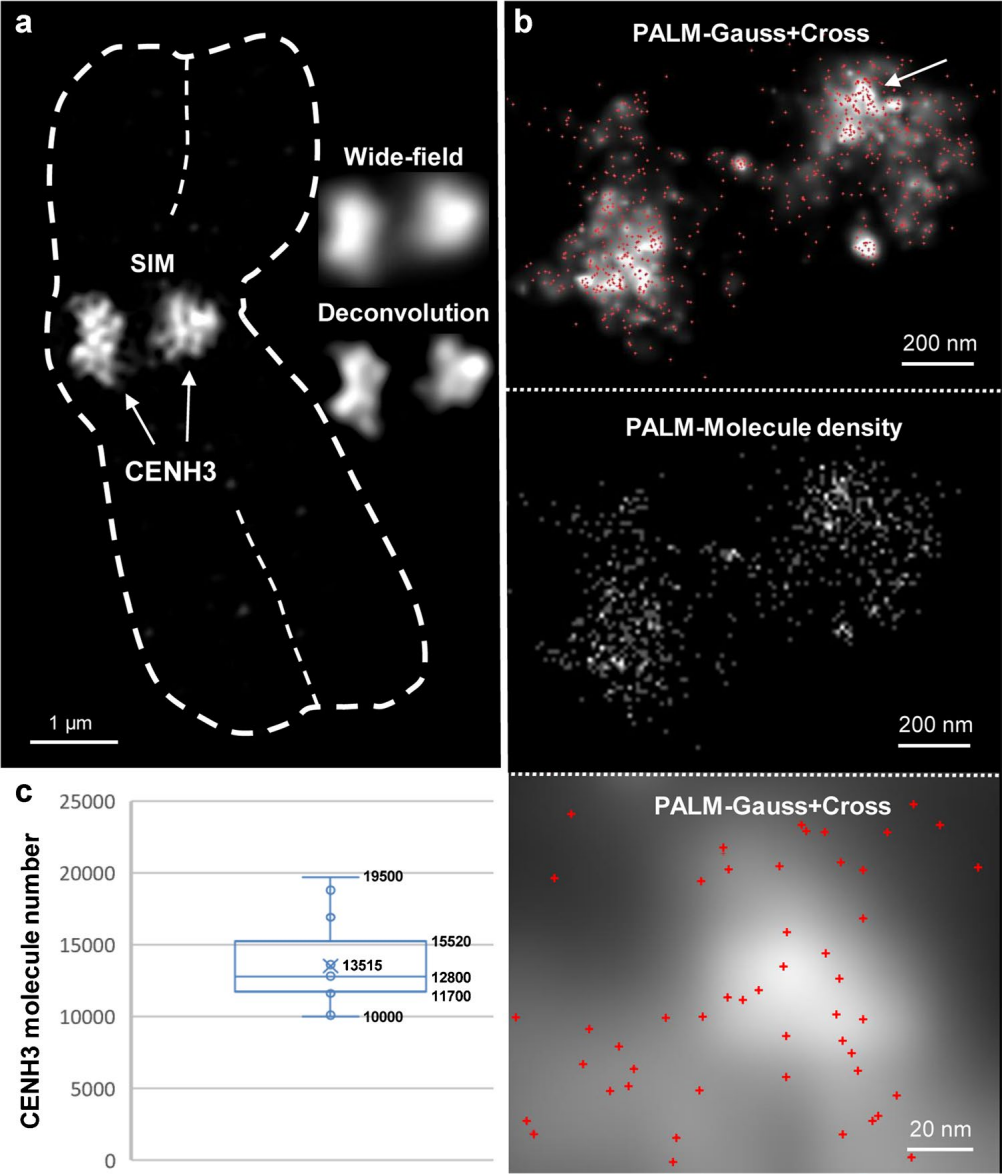
3D-SIM and 3D-PALM of CENH3-containing centromeric chromatin. **a** Both sister centromeres are labeled by CENH3 (arrows) within a barley chromosome (dashed line). SIM increases the resolution compared to widefield and deconvolution microscopy and shows the chromatin ultrastructure. **b** PALM at the same specimen. The “Gauss” and “Molecule density” presentations show the single-molecule distribution in a single slice. The crosses indicate single-molecule positions. The enlarged region (bottom) is indicated (arrow). The axial and lateral molecule localization precisions are shown in Suppl. Fig. 1. **c** Boxplot representing the CENH3 molecule number variability in 13 barley centromeres analyzed by 3D-PALM. Numbers indicate lower whisker (10,000), 25% quantile (11,700), median (12,800), mean (13,515), 75% quantile (15,520), and upper whisker (19,500)

To colocalize Topo IIα and CENH3, we immunolabeled flow-sorted barley chromosomes with specific antibodies simultaneously and applied 3D-SIM. While Topo IIα is mainly evident in the pericentromeres, CENH3 concentrates within the core of the primary constrictions and intermingles with Topo IIα-labeled chromatin (Fig. 1; Movie 3). The fluorescence signals of anti-CENH3 were detected only at centromeres, forming one CENH3-positive region per chromatid. Compared to wide-field and deconvolution microscopy, the increased resolution achieved via SIM allowed the detection of looped CENH3-labeled chromatin fibers (Fig. 3a). Besides, CENH3 chromatin may form ring-like structures) (Fig. 1), similar as found in other cereals and *Arabidopsis* (Schubert et al. 2016).

Afterward, 3D-PALM was applied on isolated chromosomes exclusively labeled with CENH3-Alexa488 antibodies (Fig. 3b, c). The labeling pattern was consistent with the SIM imaging. The distinctly localized single molecules (cross presentation) accumulate especially within bright spots of the Gauss display. A mean number of ~13,500 CENH3 molecules per chromosome, i. e., ~6750 per sister centromere, were counted. Compared to Topo IIα, the CENH3 molecule numbers varied less in the 13 barley centromeres analyzed.

3D-PALM allows the detection of single Topo IIα molecules with a lateral (XY) and axial (Z) precision of up to ~10 nm (Kubalová et al., 2021b). We achieved a similar high precision detecting the number of CENH3-containing nucleosomes. About 87% of them were localized laterally and 88% axially with a precision of 10–50 nm (Suppl. Figure 1).

Assuming an octamer structure, each centromeric nucleosome octamer of ~11 nm in diameter contains two CENH3 histones (Nechemia-Arbely et al. 2017). Our achieved PALM localization precision does not allow us to separate both CENH3 histones within a nucleosome. Consequently, each barley metaphase centromere should contain ~27,000 CENH3 molecules.

## Discussion

### 3D-PALM is useful for quantifying single molecules

In this study, we investigated the distribution and the absolute numbers of plant Topo IIα and the centromeric variant of histone H3 (CENH3) in barley metaphase chromosomes. To obtain the numbers and positions of these molecules, we combined two super-resolution microscopic techniques, SIM and PALM. Flow-sorted chromosomes were used because flat and cytoplasm-free specimens can deliver the most informative data. Two different polyclonal peptide antibodies against Topo IIα revealed the localization of this protein with a lateral and axial precisions of ~10-60 nm (Kubalová et al., 2021b). A similar precision we reached for CENH3-specific signals allowing to localize ~88% of single molecules with a distance of ~10-50 nm.

Low numbers of CENH3 molecules were detected in fission yeast with 26 by PALM (Lando et al. 2012), 84 in *Drosophila* using CENH3-EGFP fluorescence intensity measurements (Schittenhelm et al. 2010), and 25-62 in chicken DT-40 cells by SMLM or a confocal microscopy-based fluorescence ratio method (Ribeiro et al. 2010; Johnston et al. 2010). Contrary, in HeLa cells Black et al. (2007) determined at most 30,000 CENH3 molecules, i.e., ~15,000 CENH3-containing nucleosomes per centromere by using immunoblotting of extracts from randomly cycling cells with known amounts of CENH3 as quantitation standards. More recently, Bodor et al. (2014) used an indirect fluorescence method to show that human centromeres contain ~400 CENH3 molecules. This high data variability may be caused by using indirect fluorescence-based molecule counting methods containing possibly erroneous steps.

In our opinion, SMLM with its high precision to localize single molecules is currently the most reliable method to count molecules, albeit limitations apply (Shivanandan et al. 2014). It should be regarded that the number of molecules detected depends on sample integrity, staining efficiency, imaging parameters, and image processing parameters. Consequently, an exact molecule number cannot be provided. Multi-emitter algorithms might be able to single out blinking molecules, but cannot rule out the underestimation of the true molecule number (Dempsey et al. 2011; Oddone et al. 2014).

The amount of ~27,000 CENH3 molecules per centromere we detected in barley is in the range Black et al. (2007) revealed in human chromosomes and is clearly higher than determined by the other above-mentioned authors.

### Topo IIα distribution in barley differs from that in non-plant species

Here, we demonstrate that the most prominent Topo IIα fluorescence signals are present at pericentromeres, NORs of chromosomes 5H and 6H, and some telomeres of mitotic barley metaphase chromosomes. We assume that the telomeric signals of Topo IIα are chromosome-specific because both NOR-bearing chromosomes identifiable after flow-sorting always showed identical Topo IIα-labeling patterns. The high density of Topo IIα in barley pericentromeres and some telomeres is consistent with protein accumulations identified by scanning electron microscopy (Wanner and Schroeder-Reiter 2008).

At barley chromosome arms, Topo IIα is distributed in a reticulate manner. In human HeLa and Chinese hamster cells, Topo IIα, together with condensin, form a line-like protein scaffold inside each chromatid (Maeshima and Laemmli, 2003; Kireeva et al. 2004; Poonperm et al. 2015; Walther et al. 2018). These scaffold proteins were shown to be linked via bridges between the sister chromatids in pig, muntjac, and human (Chu et al. 2020). But it has also been reported that in HeLa cells, the chromatid axes occur as isolated compaction centers rather than forming a continuous line-like scaffold (Sun et al. 2018) and appear to consist of a helical structure that serves to organize chromatin loops into the metaphase chromatid (Phengchat et al. 2019). We suppose that the reticulate scaffold formation in barley may be due to different lengths of major and minor loops forming the 400 nm thick helically organized chromonema building condensed metaphase chromatids. The loop sizes fit experimental Hi-C data induced via a dispersed helical scaffold. Due to the intermingling of ~80 nm lower-order chromatin fibers, the helical chromonema structure is not visible by 3D-SIM on homogeneously stained chromatids but via differential oligo-FISH labeling (Kubalová et al. 2021a).

One Topo IIα enzyme for every 20-50 kb of DNA was estimated to be present in mitotic HeLa metaphase chromosomes (Gasser et al. 1986; Fukui and Uchiyama 2007). In barley chromosome arms, we determined ~27,400 Topo IIα molecules, ~28% of them around centromeres. The genome size of barley containing seven chromosomes is 4.65 Gb DNA (Monat et al. 2019). This corresponds to 664.3 Mb per chromosome. Thus, one molecule of Topo IIα resides at approximately every 24 kb (664.3 Mb /27,400 = 24 kb) of the whole barley chromosome, a value similar to that found for HeLa cells. Due to the accumulation of Topo IIα (~7,700 molecules) in the barley pericentromeres spanning ~100 Mb DNA (Monat et al. 2019), we assume one molecule per 13 kb (100 Mb/7700 =13 kb), and along chromosome arms a lower density with one molecule every 32.5 kb (564.3 Mb/17,400=32.5 kb).

Besides the detection of the accumulation of Topo IIα in pericentromers, NORs and telomeres, and a less prominent amount along chromosome arms, PALM revealed the clustering of Topo IIα in these regions, possibly representing chromatin fiber looping centers. Given the role of Topo IIα in chromosome condensation and its reticular distribution along the chromosomal arms, it would be of interest to apply PALM to condensins, the key components in chromosome organization (Hirano 2012). Walther et al. (2018) detected by fluorescence correlation spectroscopy ∼195,000 condensin I and ∼35,000 condensin II complexes in HeLa chromosomes. Determining the number and distribution of condensins also in barley chromosomes would improve the understanding of the mitotic condensation process.

### Topo IIα and CENH3 are part of the (peri)centromeric chromatin

Besides epigenetic DNA and histone modifications (Vos et al. 2006; Gieni et al. 2008; Achrem et al. 2020), cohesin, condensin, and SMC5/6 complexes, the main components to organize (peri)centromeres are Topo IIα and CENH3 (Wang et al. 2010; Gomez et al. 2013, 2014; Lawrimore and Bloom 2019a, b). Like in yeast and frog (Ryu et al. 2015; Edgerton et al. 2016; Yoshida et al. 2016; Zhang et al. 2020), barley Topo IIα localizes to the centromeres of metaphase chromosomes.

A positive correlation exists between kinetochore and chromosome size, and the adequate number of attached microtubule spindle fibers are important for correct chromosome segregation during cell division. Larger chromosomes require more microtubules, and thus larger kinetochores to move them with the same velocity as small ones (Nicklas 1965; Plačková et al. 2022). The microtubule-binding capacity increases with kinetochore size in Indian muntjac chromosomes (Drpic et al. 2018) and in rat-kangaroo PtK1 cells, and it was demonstrated that the chromosome size determines the number of microtubules (McEwen et al. 1998). In grass species, the anti-CENH3 signal size is strongly correlated with genome size. Species with large genomes and few chromosomes have the largest centromeres (e.g., rye), while species with small genomes and many chromosomes have the smallest centromeres (e.g., rice) (Zhang and Dawe 2012). Although not as obvious as between species, a positive correlation between kinetochore size and chromosome size was also observed in human (Irvine et al. 2004) and maize (Wang et al. 2021), and within bimodal karyotypes as demonstrated for *Agavoideae* species (Plačková et al. 2022).

We determined ~27,000 CENH3 molecules per centromere for the relatively large barley chromosomes. For comparison, it will be interesting to elucidate the CENH3 amount in small chromosomes by PALM/SMLM.

## Materials and methods

### Plant material, chromosome isolation, and specimen preparation

Barley metaphase chromosomes (*Hordeum vulgare* L. cv. Morex) were sorted according to Lysák et al. (1999). Briefly, a chromosome suspension was prepared from synchronized primary roots meristems. Chromosomes were DAPI-stained, immediately analyzed, and flow-sorted using a FACSAria II SORP flow cytometer and sorter (BD Bioscience, San Jose, CA, USA). Five thousand chromosomes were sorted into 15 μl of PRINS buffer supplemented with 2.5% sucrose (10 mM TRIS, 50 mM KCl, 2 mM MgCl_2_.6H_2_O, 2.5% sucrose; pH 8) onto high precision coverslips (Paul Marienfeld GmbH & Co. KG, Lauda-Königshofen, Germany). Before immunolabeling, the coverslips were stored at −20 °C.

### Indirect immunostaining

Before immunolabeling, coverslips were washed twice with 1xPBS keep in one line for 5 min at room temperature (RT) and incubated with blocking solution (5% BSA, 0.03% Triton X-100, 1×PBS) for 1.5 h at RT. Peptide Topo IIα (rb12 and gp13) (Kubalová et al. 2021b) and rabbit anti-grassCENH3 (Nagaki et al. 2004; Houben et al. 2007) antibodies were diluted 1:100 and 1:10,000, respectively, in antibody solution (1% BSA, 0.01% Triton X-100, 1 × PBS), and incubated overnight at 4 °C. Grass-CENH3 antibodies detect both α and βCENH3 of barley (Ishii et al. 2015).

Next, coverslips were washed with 1×PBS (three times, 5 min each) at RT and incubated with secondary donkey anti-rabbit Alexa488 (1:200, #711-545-152 Jackson ImmunoResearch) and goat anti-guinea pig Alexa488 (1:200, # A11073 Invitrogen) antibodies for 1 h at 37 °C. For colocalization with Topo IIα, CENH3 was labeled with Cy3-conjugated anti-rabbit IgG (Dianova). Subsequently, coverslips were washed in 1×PBS (three times, 5 min each) at RT and immediately dehydrated in an ethanol series (70%, 85%, and 100%), each step 2 min. Afterward, the coverslips were air-dried and subjected to microscopy.

### Microscopy

The fluorescence signals of Topo IIα and CENH3 were imaged by wide-field (WF), deconvolution (DCV) of WF, and super-resolution 3D-SIM, using an Elyra PS.1 microscope system equipped with a 63×/1.4 Oil Plan-Apochromat objective and the software ZENBlack (Carl Zeiss GmbH). Images were captured separately for DAPI and Alexa488 using 405 nm and 488 lasers for excitation and appropriate emission filters. Reconstruction of SIM images was done with the ZENBlack software structured illumination processing module. 3D-PALM was performed with the 488 laser and the images were processed with the ZENBlack software PALM processing module. The localization precision in 3D-PALM was calculated via simulations of the experimental point-spread function (Weisshart et al. 2016; Kubalová et al. 2021b). The localization precision is the standard deviation of the data fit. Therefore, it describes the certainty of the localized position or likewise the area within which the molecule is positioned with high likelihood. Determining the resolution is not straightforward and would require the spacing of the labeled molecules. If the spacing of molecules is at least twice as fine as the localization precision, the latter represents according to the Nyquist criterion the resolution. Otherwise, the resolution is twice the spacing. As spacing normally is not known, one has to resort to taking the profile between two structures as described in Kubalová et al. (2021b) to determine the resolution at a specific site. The resolution can be quite different in various areas of the image.

3D rendering of SIM and PALM image stacks to produce movies was performed with the Imaris 9.7 software (Bitplane).

## Supplementary Information

Suppl. Figure 1. **Lateral and axial 3D-PALM localization precision of CENH3 molecules achieved within barley centromeres. a)** Diagrams showing the 3D-PALM XY (lateral)- and Z (axial)-localization precision of all CENH3-labelled molecules detected in both chromatids of a barley metaphase chromosome centromere (see Figure 2). The red bars frame the percentage of molecules that were localized with a precision of 10-50 nm. **b)** Distribution of the lateral (left) and axial (right) localization precisions of CENH3 molecules in 10 analysed centromeric regions displayed as a boxplot. These single measurements were used to calculate the averaged values shown in the right boxplot. The numbers below indicate the mean ± standard deviation of the molecules in all 10 centromeres. (PNG 229 kb)

High Resolution Image (TIF 939 kb)

Movie 1: Running through a SIM image Z stack of the chromosome shown in Figure 2b. (AVI 34.8 MB)

Movie 2: Rotation of the SIM image Z stack of the chromosome shown in Figure 2b. (MP4 4.33 MB)

Movie 3: Rotation of the SIM image Z stack of the enlarged centromeric region shown in Figure 1. The intermingling of Topo IIα (green) and CENH3 (red) is clearly visible. Whole chromatin was counterstained by DAPI (blue). (MP4 14.9 MB)

Movie 4: Running through a PALM-Gauss image Z stack of the chromosome shown in Figure 2b. (AVI 40603 kb)

## Data Availability

There are no additional data and material available.
