# Supplementary figures and images for: Super-resolution microscopy reveals the number and distribution of topoisomerase IIα and CENH3 molecules within barley metaphase chromosomes
Source: Chromosoma. 2023 Jan 31;132(1):19–29. doi: 10.1007/s00412-023-00785-8 (PMC9981516; doi:10.1007/s00412-023-00785-8)

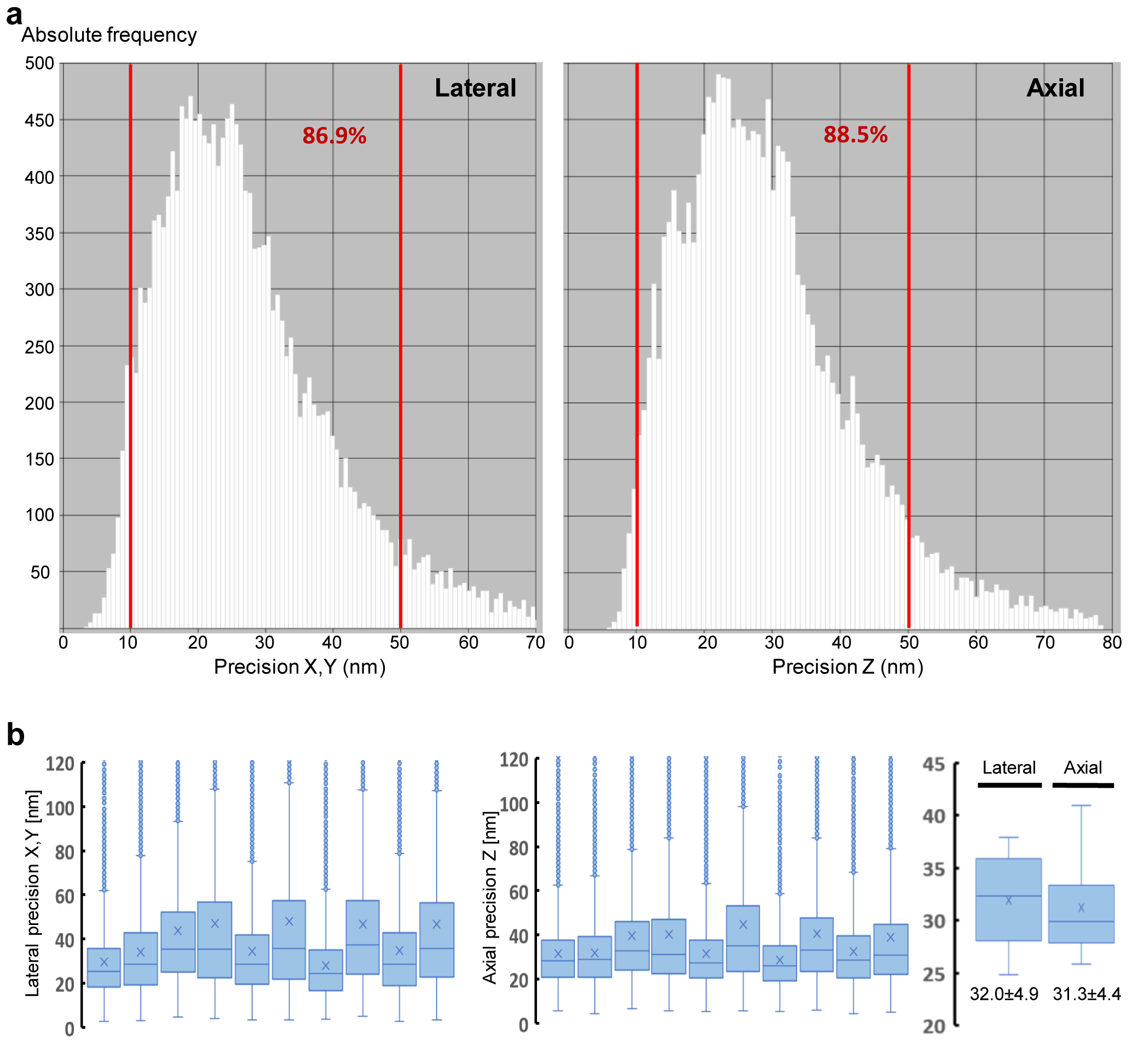

Supplement: Supplementary file 1 — Lateral and axial 3D-PALM localization precision of CENH3 molecules achieved within barley centromeres. a) Diagrams showing the 3D-PALM XY (lateral)- and Z (axial)-localization precision of all CENH3-labelled molecules detected in both chromatids of a barley metaphase chromosome centromere (see Figure 2). The red bars frame the percentage of molecules that were localized with a precision of 10-50 nm. b) Distribution of the lateral (left) and axial (right) localization precisions of CENH3 molecules in 10 analysed centromeric regions displayed as a boxplot. These single measurements were used to calculate the averaged values shown in the right boxplot. The numbers below indicate the mean ± standard deviation of the molecules in all 10 centromeres. (PNG 229 kb) [file 412_2023_785_Fig4_ESM.png]

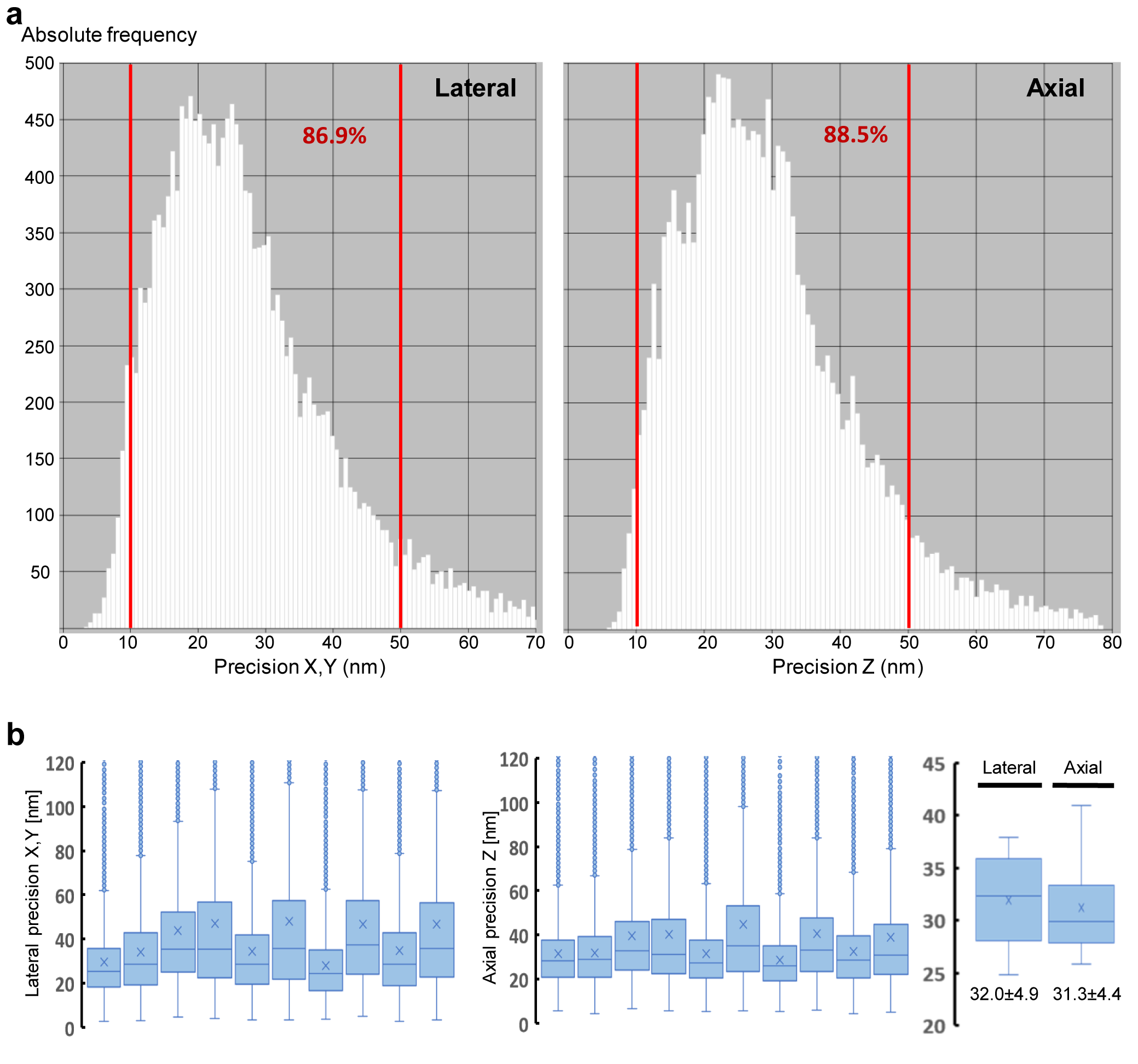

Supplement: Supplementary file 2 — High Resolution Image (TIF 939 kb) [file 412_2023_785_MOESM1_ESM.tif]
